# Effects of CRMP2 DNA Methylation in the Hippocampus on Depressive-Like Behaviors and Cytoskeletal Proteins in Rats

**DOI:** 10.3389/fncel.2021.644663

**Published:** 2021-03-17

**Authors:** Dan Xiang, Siqi Sun, Gaohua Wang, Zhongchun Liu

**Affiliations:** ^1^Department of Psychiatry, Renmin Hospital of Wuhan University, Wuhan, China; ^2^Institute of Neuropsychiatry, Renmin Hospital, Wuhan University, Wuhan, China

**Keywords:** depression, CRMP2, DNA methylation, CUMS, microtubule dynamics

## Abstract

Chronic stress appears to alter DNA methylation and DNA methyltransferases (DNMTs) in brain regions related to emotion. Collapsin response mediator protein-2 (CRMP2) mediates the development of depression by regulating microtubule dynamics. In this study, rats were subjected to chronic unpredictable mild stress (CUMS). At the end of the CUMS procedure, normal saline or fluoxetine was administered to the rats. Moreover, normal saline or the 5-aza-2’-deoxycytidine (5-aza) was administered to the hippocampal CA1 region of the rats. Behavioral tests were performed to evaluate the depressive-like phenotypes. The CRMP2 DNA methylation levels and cytoskeletal microtubular system-related biomarkers were detected by several molecular biology techniques. The results showed that the rat model of depression was successfully established by exposure to CUMS, and fluoxetine treatment exerted an antidepressant-like effect. We observed the upregulation of DNMT1 and DNMT3a in the hippocampus of stressed rats. CUMS induced a decrease in CRMP2 expression and an increase in phosphorylated CRMP2 (pCRMP2) expression in the hippocampus of rats. The rate of DNA methylation in the CpG island of the CRMP2 promoter region in the hippocampus of stressed rats was significantly higher than that in control rats. Moreover, CUMS significantly decreased the interaction between CRMP2 and α-tubulin and decreased the microtubule dynamics. Chronic fluoxetine treatment reversed these changes. Also, hypomethylation induced by 5-aza injection into the hippocampal CA1 region caused antidepressant-like effects and increased CRMP2 expression and microtubule dynamics. These results suggested that CRMP2 DNA methylation may be involved in regulating the cytoskeletal microtubular system and mediating depressive-like behaviors.

## Introduction

As a severe psychiatric disorder, depression is characterized by a depressed mood or a marked loss of interest or pleasure for most of the day. Depression is a common illness worldwide, and more than 300 million people are currently depressed. Depression has substantial adverse effects on personal health, with high recurrence and mortality. Depression is linked to cerebrovascular disease, and depressed patients are at higher risk of stroke, with an enormous burden for patients, families, and society as a whole (Kolovos et al., 2017; Winter et al., 2018). Although various treatments are effective against depression, some patients are resistant to the currently available treatments. The underlying mechanisms that cause depression are still unclear. Therefore, it is necessary to identify the mechanisms underlying depression.

Collapsin response mediator protein-2 (CRMP2), which has also been identified as dihydropyrimidinase-like 2 (DPYSL2), belongs to the CRMP family that includes 5 homologs (CRMP1-5). CRMP2 is enriched in brain areas that retain plasticity and neurogenesis, such as the hippocampus, olfactory bulb, and cerebellum (Inagaki et al., 2001). CRMP2 binds to tubulin and is phosphorylated by various kinases, which regulates its activity (Yamashita and Goshima, 2012). Microtubules are crucial cytoskeletal constituents in growing neurites and axons. Several studies have suggested that CRMP2 assists microtubule assembly together with the tubulin heterodimer (Fukata et al., 2002; Niwa et al., 2017). CRMP2 binds to the tubulin heterodimer, and upon phosphorylated CRMP2 (pCRMP2) by various kinases, the binding affinity of CRMP2 to tubulin weakens. CRMP2 is a microtubule-associated protein that regulates the dynamic process of microtubule assembly (Gu and Ihara, 2000). Microtubule dynamics can be identified by analyzing the stable form, acetylated α-tubulin (Acet-tubulin), and the dynamic form, tyrosinated α-tubulin (Tyr-tubulin), which are associated with neuronal plasticity (Bianchi et al., 2006). Consistent with the role of CRMP2 in neuronal functions, CRMP2 has shown to be implicated in several neuropsychiatric disorders, including cerebral ischemia, schizophrenia, and depression (Quach et al., 2015). CRMP2 levels are decreased in the brains of patients with depression (Johnston-Wilson et al., 2000). Additionally, the proteomic analysis showed that chronic treatment with the antidepressive agents venlafaxine or fluoxetine increased CRMP2 levels in the rat hippocampus (Khawaja et al., 2004). In the previous research of our group, Wu et al. have confirmed the effect of CRMP2-mediated neuronal plasticity in depression induced by chronic stress (Wu et al., 2018). However, the molecular mechanisms by which CRMP2 contributes to the pathogenesis of depression remain to be elucidated.

Exposure to adverse environmental events is one of the strongest risk factors for depression. Mechanisms of epigenetic modification include DNA methylation, chromatin conformational changes, long noncoding RNAs, and histone modifications, and these mechanisms can modulate gene expression in response to the environment (Mahgoub and Monteggia, 2013). DNA methylation is the most stable form of epigenetic modification, and several lines of evidence suggest a critical role for this modification in depression (Chen et al., 2017; Lisoway et al., 2018). DNA methylation is accomplished by DNA methyltransferases (DNMTs), which catalyze the addition of a methyl group to the cytosine in the 5’ position of cytosine-phosphate-guanine sites. The DNMTs family mainly includes DNMT1, DNMT3a, and DNMT3b (Kader et al., 2018). DNMT1 is predominantly associated with the maintenance of DNA methylation, and DNMT3a and DNMT3b are more strongly associated with de novo methylation (Okano et al., 1999). Candidate genes that undergo DNA methylation, such as brain-derived neurotrophic factor (BDNF), NR3C1 (encoding the glucocorticoid receptor), SLC6A4 (encoding the serotonin transporter), have been regarded as potential biomarkers of depression. Previous studies demonstrated that increased DNA methylation in the promoter region of BDNF was associated with the pathophysiology of depression (Januar et al., 2015). A study has shown that animals subjected to early life stress were associated with a specific increase in DNA methylation levels of the exon 1F NR3C1 gene (Weaver et al., 2004). Additionally, several studies have demonstrated an increase in DNA methylation of SLC6A4 in depression (Philibert et al., 2008; Zhao et al., 2013). CRMP2 has been proposed to be a candidate gene for the treatment of depression. In our previous study, the results showed a reduction in CRMP2 expression in the hippocampus of stressed rats, which may correlate with a significant increase in the DNA methylation levels of the CRMP2 promoter region (Xiang et al., 2020). Moreover, several studies suggested the important role of DNMTs in regulating depressive-like behaviors (Ignácio et al., 2017; Shen et al., 2019). Systemic or intrahippocampal administration of DNMTs inhibitors induces antidepressant-like effects in animals subjected to the forced swimming test (FST) and open field test (OFT; Sales et al., 2011). As described above, these studies support the hypothesis that DNA methylation is an important epigenetic modification involved in the pathogenesis of depression.

Despite these pieces of evidence, little is known about the role of CRMP2 DNA methylation in the etiology of depression. In this study, chronic unpredictable mild stress (CUMS) was used to establish a model of depression. The sucrose preference test (SPT), FST, and OFT were used to evaluate depressive-like behaviors. We investigated how CUMS and fluoxetine treatment affect DNMTs expression and CRMP2 DNA methylation in the hippocampus of rats. We also detected CRMP2, pCRMP2, and α-tubulin isoform expression in the hippocampus of rats to elucidate the underlying mechanisms by which CRMP2 is involved in the pathological processes of depression. Hippocampus is the most commonly studied brain region in depression research, and it is part of the limbic structures, which are associated with emotional responses. In this study, we focused on the role of the hippocampal region, as neural plasticity in this brain region has been related to depressive-like behavior.

We investigated the effect caused by injecting the DNMTs inhibitor 5-aza-2’-deoxycytidine (5-aza) into the hippocampal CA1 regions of rats. The effect of 5-aza on the levels of CRMP2, pCRMP2, and α-tubulin isoforms in the hippocampal CA1 region was also assessed to further explore the mechanism by which CRMP2 is involved in the pathogenesis of depression.

## Materials and Methods

### Animals

Adult male Sprague–Dawley rats weighing 180–200 g were purchased from the Company of Experimental Animals of Hunan Slack King (Hunan, China). Before the experiment, the rats were housed under laboratory conditions for seven days to adapt to the new environment. The rats were housed at 22 ± 2°C with a 12-h light/12-h dark schedule with free access to food and water. All the procedures and animal experiments of this study were performed in agreement with the guidelines outlined in the legislation of the P.R. China regarding the ethical use of laboratory animals and approved by the Institutional Animals Care Committee of Renmin Hospital of Wuhan University.

### Experimental Design

**Figure 1:**
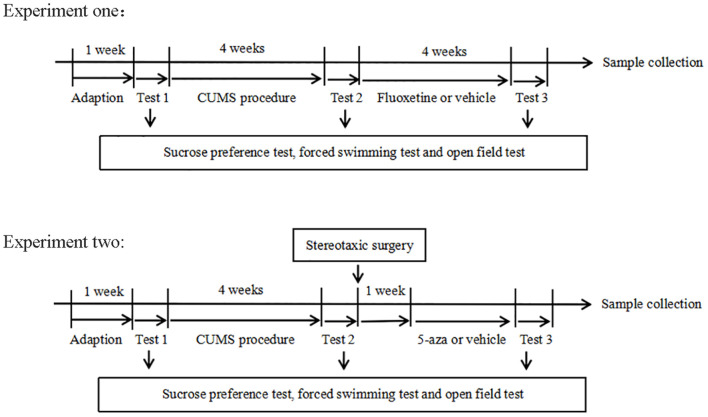
Experimental procedures.

The experimental design is shown in Figure 1. Experiment one: The rats were randomly divided into three groups: the control group (*n* = 20), the CUMS group (*n* = 20), and the fluoxetine group (*n* = 20). In both the CUMS and fluoxetine groups, the rats were subjected to CUMS daily for four weeks. At the end of the CUMS procedure, the rats in the CUMS group were given an intraperitoneal injection of saline vehicle, and the rats in the fluoxetine group were given an intraperitoneal injection of fluoxetine (10 mg/kg) for 4 weeks. The rats in the control group did not receive any stimuli throughout the entire procedure. Behavioral tests were used to evaluate the effects of stress and fluoxetine on anhedonia and activity. We investigated the effects of CUMS and fluoxetine treatment on DNA methylation in the CRMP2 promoter and DNMTs expression. Additionally, we analyzed the protein expression of CRMP2 and pCRMP2 and the expression of α-tubulin isoforms, which are thought to reflect microtubule dynamics. We also explored the effect of chronic stress and antidepressant treatment on the interaction between CRMP2 and α-tubulin.

Experiment two: a total of 20 rats were subjected to the CUMS procedure and were further divided into two groups (*n* = 10/group): the CUMS + normal saline group (CUMS + Nacl group) and the CUMS + 5-aza group. Intrahippocampal injections of the saline vehicle were administered to rats in the CUMS + Nacl group. 5-aza was diluted in normal saline and administered into the hippocampus of the rats in the CUMS + 5-aza group. Behavioral tests were used to evaluate the effects of 5-aza on depressive-like behaviors. We examined the global DNA methylation levels in the hippocampal CA1 region of the rats treated with 5-aza. Moreover, the expression of CRMP2, pCRMP2, and α-tubulin isoforms in the hippocampal CA1 region was analyzed, and the interaction between CRMP2 and α-tubulin was examined in the rats treated with 5-aza.

### CUMS Procedure

The CUMS procedure was performed as previously described (Xiang et al., 2019). The CUMS procedure consisted of a series of seven different stressors: food deprivation for 24 h; water deprivation for 24 h; cage tilted at 45° for 24 h; swimming in 4° ice water for 5 min; tails clamped for 3 min; exposure to damp sawdust for 24 h and inversion of the light/dark cycle for 24 h. The same stressor was not applied on any two consecutive days.

### Stereotaxic Surgery and Hippocampal CA1 Region Administration

The rats subjected to stereotaxic surgery were anesthetized with sodium pentobarbital (25 mg/kg) and fixed in a stereotaxic frame. The rats were bilaterally implanted with stainless steel guide cannulae aimed at the hippocampal CA1 region (coordinates: 3.5 mm posterior to the bregma, ±2.3 mm lateral, 2.6 mm from the cortical surface). Matched stylets were inserted into the guide cannulae to prevent obstruction. After the surgery, the rats were injected with penicillin for 3 days to prevent infection.

Seven days after the surgery, hippocampal CA1 administration was performed with a microsyringe. 5-aza (Sigma–Aldrich, A3656; 100 nmol/0.5 μl) was dissolved in normal saline and injected into the hippocampal CA1 region (0.5 μl per side). 5-aza was slowly infused over 1 min, and the microsyringe was maintained in the guide cannula for 2 min to prevent backflow. Rats received three injections of 5-aza or saline vehicle, and the third injection was given 1 h before the behavioral tests.

### Behavioral Tests

The SPT, OFT, and FST were performed in this study. The SPT was performed as previously described. Before the test, the rats were trained to consume 1% sucrose solution. After the training, the rats were deprived of water for 24 h and then offered a bottle of 1% sucrose solution and a bottle of water for the next 24-h period. Sucrose consumption was measured by comparing the weights of the bottles before and after the test. The locomotor activity of the rats was assessed using the OFT. In the test, one rat was placed in a square arena (100 × 100 × 35 cm) and observed using a video tracking system (Ethovision XT 11.5) for 5 min. The total distance traveled, the average velocity, and the rearing frequency were recorded. The apparatus was cleaned with a 75% alcohol solution after each test. In the FST, a rat was placed in a glass cylinder (30 cm diameter × 40 cm height) that was filled with water to a height of 28 cm and maintained at 25°C. The rat was forced to swim for 6 min, and the immobility time was recorded during the final 4 min. The immobility time in the FST was regarded as the time the rats spent floating in the water without struggling or exhibiting only slight movement to keep their heads above the water.

### Bisulfite Sequencing PCR (BSP)

Genomic DNA was extracted from hippocampal tissue using a genomic DNA extraction kit (Tiangen, DP304), and bisulfite conversion was performed using an EpiTect Bisulfite Kit (Qiagen, 59104) according to the manufacturer’s protocol. The bisulfite-modified DNA samples were amplified by bisulfite sequencing PCR with primers specific to the CRMP2 gene promoter. The promoter of the CRMP2 gene is generally considered to be a 2,000-bp sequence upstream of the transcription start site. The primers were designed by MethPrimer software. The BSP primers were as follows: F: 5’-TTTGTATTGTAGATGAAGTA TTTGGG-3’; R: 5’-AACAATAAAAACCTTAATTCCAATC-3’. The PCR conditions were as follows: 95°C for 30 s, 40 cycles of 95°C for 5 s, 50°C for 30 s, and 72°C for 30 s. The PCR products were separated by 2% agarose gel electrophoresis, purified with a gel extraction kit (Cwbio, CW2302M), and then cloned into the pEASY-T1 cloning vector (TransGen, CT101–01). The recombinant plasmids were transformed into *E. coli* DH5α. At least ten clones of each sample were randomly chosen and sequenced by the Wuhan Tianyi Huiyuan Company.

### 5-mC DNA ELISA

Genomic DNA was extracted from the hippocampal CA1 region using the TIANamp Genomic DNA kit (TIANGEN Biotech, DP304) following the manufacturer’s protocol. The DNA samples were quantified by a Nanodrop, and the global DNA methylation levels were determined by measuring the levels of 5-mC using a 5-mC DNA ELISA Kit (Zymo Research, D5325) following the manufacturer’s protocol. One hundred nanograms of genomic DNA was measured per well, and independent samples were run in triplicate. The absorbance was analyzed at 420 nm using a microplate reader. The mean percentages of 5-mC were calculated using the second-order regression equation of the standard curve as described in the manufacturer’s protocol.

### Western Blot

The proteins from the hippocampus were separated using a 10-12% SDS-PAGE gel and transferred to PVDF membranes. The membranes were blocked with 5% fat-free milk for 1 h at room temperature and incubated with primary antibodies overnight at 4°C; the primary antibodies included anti-CRMP2 (1:20,000; Abcam, ab129082); anti-pCRMP2 (Thr514 site) (7 μl: 5 ml; Abcam, ab85934); anti-DNMT1 (1:5,000); anti-DNMT3a (1:5,000); anti-DNMT3b (1:5,000); anti-α-tubulin (1:5,000; Abcam, ab7291); anti-Tyr-Tubulin (1:2,500; Sigma, T9028); anti-Acet-Tubulin (1:200; Santa Cruz Biochemistry, sc-23950); and anti-GAPDH (1:5,000; Beyotime, AF1186). After washing three times, the membranes were incubated with the appropriate horseradish peroxidase (HRP)-conjugated secondary antibodies (Beyotime, 1:5,000) at room temperature for 1 h. Then, the membranes were visualized by an ECL chemiluminescent detection kit (Beyotime, P0018S), and the intensities of the protein bands were calculated by ImageJ software. The relative expression of the proteins was normalized to that of GAPDH.

### Co-immunoprecipitation Analysis

Total protein (500 μg) was incubated with anti-CRMP2 (1:50; Abcam, ab129082) or anti-α-tubulin (1:50) (Abcam, ab7291) overnight at 4°C. An equal amount of protein was incubated with mouse IgG (Beyotime, A7028) or rabbit IgG (Beyotime, A7016) as negative controls. The protein samples were loaded as the input control. Protein A + G agarose (Beyotime, P2012) was washed with PBS three times and mixed with the samples. The mixtures were incubated at 4°C for 2 h. The beads were then washed with PBS three times. The immunoprecipitates were subsequently subjected to 12% SDS-PAGE and analyzed *via* western blot using anti-α-tubulin (1:5,000) or anti-CRMP2 (1:20,000) antibodies. The protein bands were visualized using an ECL chemiluminescent detection kit (Beyotime, P0018S).

### Statistical Analysis

Statistical analysis was performed using SPSS 20.0 software, and the data are expressed as the mean ± standard error of the mean (SEM). The normality of the data distribution was analyzed. For differences between the two groups, Student’s *t*-test was used to determine statistical significance. For multiple comparisons, ANOVA followed by an LSD *post hoc* test was used. Differences were considered to be statistically significant at *P* < 0.05.

## Results

### Effect of CUMS and Fluoxetine Treatment on Depressive-Like Behaviors

**Figure 2:**
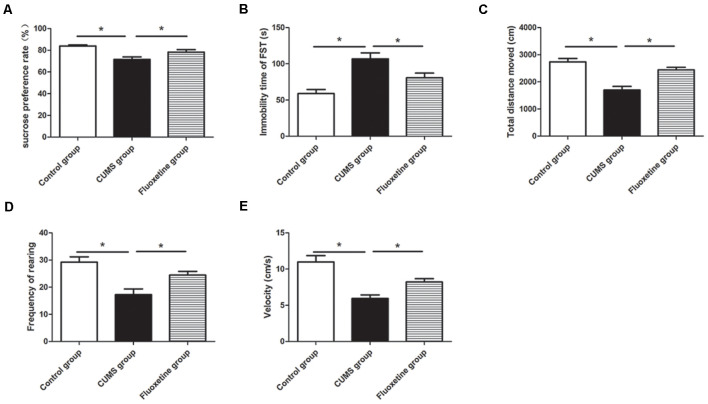
Effect of chronic unpredictable mild stress (CUMS) and fluoxetine treatment on depressive-like behaviors: sucrose preference rate in the sucrose preference test (SPT; **A**), immobility time in the FST **(B)**, total distance traveled **(C)**, rearing frequency **(D)**, and velocity **(E)** in the open field test (OFT). *P < 0.05.

CUMS-induced behavioral changes were measured using the SPT, OFT, and FST. There were significant differences in the sucrose preference, distance traveled, rearing frequency and velocity in the OFT among the three groups (sucrose preference: *F* = 39.24, *P* < 0.05, Figure 2A; distance traveled: *F* = 20.34, *P* < 0.05, Figure 2C; rearing frequency: *F* = 13.99, *P* < 0.05, Figure 2D; velocity: *F* = 16.13, *P* < 0.05, Figure 2E). Compared with the rats in the control group, the rats in the CUMS group displayed significantly decreased sucrose preference (*P* < 0.05), distance traveled (*P* < 0.05), rearing frequency (*P* < 0.05), and velocity (*P* < 0.05). Treatment with fluoxetine for 4 weeks significantly alleviated depressive behaviors in the rats. Fluoxetine administration significantly reversed the decrease in sucrose preference (*P* < 0.05), distance traveled (*P* < 0.05), rearing frequency (*P* < 0.05), and velocity (*P* < 0.05) in the stressed rats. Moreover, there were significant differences in immobility time in the FST among the three groups (*F* = 16.70, *P* < 0.05, Figure 2B). Compared with the rats in the control group, after exposure to CUMS for 4 weeks, the rats in the CUMS group exhibited significantly increased immobility times in the FST (*P* < 0.05), and the rats treated with fluoxetine exhibited significantly decreased immobility times compared to the rats exposed to CUMS alone (*P* < 0.05).

### Effect of CUMS and Fluoxetine Treatment on DNA Methylation in the CRMP2 Promoter

**Figure 3:**
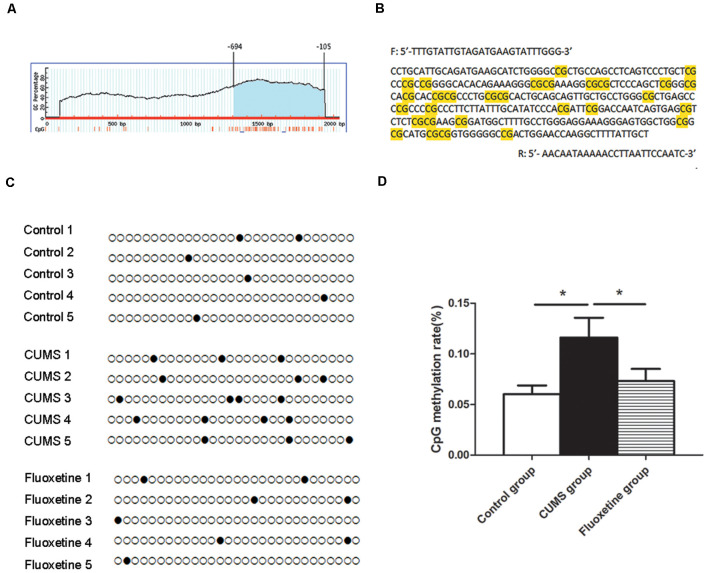
Effect of CUMS and fluoxetine treatment on DNA methylation in the CRMP2 promoter: 1 CpG island region located from −694 to −105 with 29 CpG sites was identified based on the CRMP2 promoter sequence **(A)**; BSP primer targeted the CpG islands in the promoter region of CRMP2 was designed, and the CpG dinucleotides are highlighted **(B)**; the methylation levels of the promoter region of the CRMP2 gene in the hippocampus **(C,D)**, black circles represent methylated CpG sites, and white circles represent unmethylated CpG sites. *P < 0.05.

Using MethPrimer online software, 1 CpG island region located from −694 to −105 with 29 CpG sites was identified based on the CRMP2 promoter sequence (Figure 3A). The CpG island sequence in the CRMP2 promoter is shown in Figure 3B, and the CG dinucleotides are highlighted. CpG islands are regions of genomic DNA enriched for CG dinucleotides. Treatment of genomic DNA with bisulfite resulted in the conversion of unmethylated cytosine to uracil, however, the methylated cytosine remains unchanged. The DNA methylation rate was calculated as the “CG” sites divided by the sum of the “CG” + “TG” sites. In the CpG island region, the BSP results showed that the DNA methylation of CRMP2 in the hippocampus of rats exposed to CUMS was increased compared with that in the hippocampus of control rats, and fluoxetine treatment significantly decreased the DNA methylation in the CRMP2 promoter (DNA methylation rate: control group: 6.0 ± 0.9%; CUMS group: 11.6 ± 1.9%; fluoxetine group: 7.3 ± 1.2%; *F* = 4.27, *P* < 0.05, Figures 3C,D).

### Effect of CUMS and Fluoxetine Treatment on DNMT Expression

**Figure 4:**
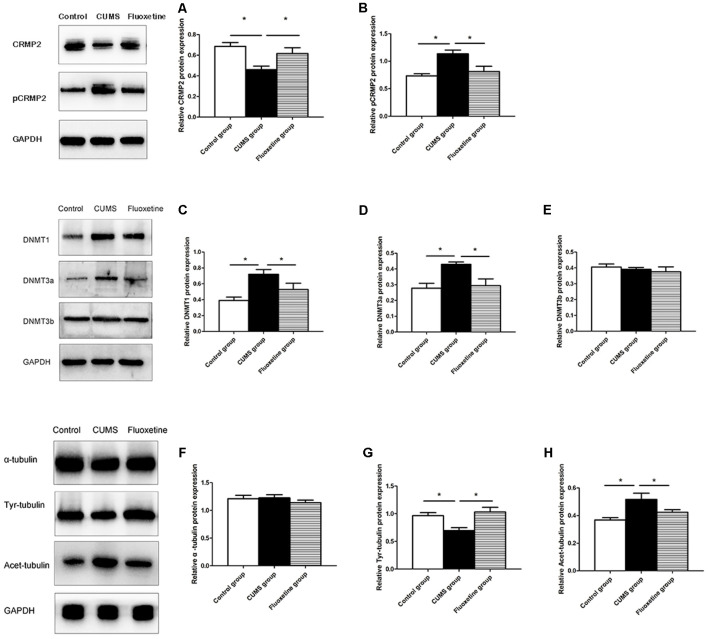
Effect of CUMS and fluoxetine treatment on CRMP2 **(A)**, pCRMP2 **(B)**, DNMTs (DNMT1: **C**; DNMT3a: **D**; DNMT3b: **E**), and α-tubulin isoform (α-tubulin: **F**; Tyr-tubulin: **G**; Acet-tubulin: **H**) expression in the hippocampus of rats. *P < 0.05.

Western blotting was conducted to evaluate the effect of CUMS and fluoxetine treatment on the expression of DNMTs. Compared with that in the control group, the protein expression of DNMT1 and DNMT3a was significantly increased in the hippocampus of the CUMS group, and fluoxetine administration significantly downregulated the protein expression of DNMT1 and DNMT3a (DNMT1 *F* = 7.39, *P* < 0.05, Figure 4C; DNMT3a *F* = 6.74, *P* < 0.05, Figure 4D; DNMT3b *F* = 0.46, *P* > 0.05, Figure 4E).

### Effect of CUMS and Fluoxetine Treatment on CRMP2, pCRMP2, and α-Tubulin Isoform Expression

Compared with that in the control group, CRMP2 protein expression was decreased in the hippocampus of the CUMS group, and fluoxetine treatment increased CRMP2 protein expression (*F* = 7.11, *P* < 0.05, Figure 4A). The pCRMP2 protein expression in the hippocampus of rats exposed to CUMS was significantly higher than that observed in the control rats and rats treated with fluoxetine (*F* = 9.07, *P* < 0.05, Figure 4B). We further explored the α-tubulin, Tyr-Tubulin, and Acet-Tubulin protein expression in the three groups. The results showed that the α-tubulin protein expression in the hippocampus of the rats in each group was not significantly different (*F* = 0.68, *P* > 0.05, Figure 4F). Compared with those in the control group, Tyr-tubulin protein expression was downregulated and Acet-tubulin protein expression was upregulated in the hippocampus of the CUMS group, and fluoxetine treatment reversed these changes (Tyr-tubulin: *F* = 7.47, *P* < 0.05, Figure 4G; Acet-tubulin: *F* = 6.41, *P* < 0.05, Figure 4H).

### Effect of CUMS and Fluoxetine Treatment on the Interaction Between CRMP2 and α-Tubulin

**Figure 5:**
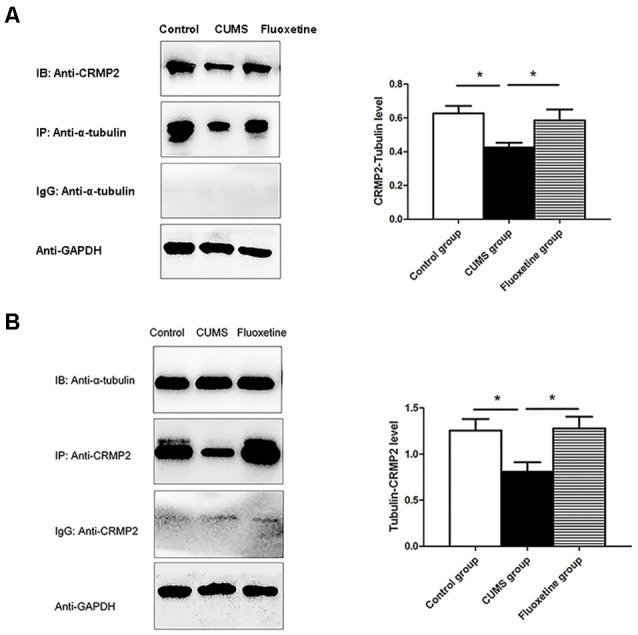
Effect of CUMS and fluoxetine treatment on the interaction between CRMP2 and α-tubulin **(A,B)**. *P < 0.05.

To investigate the interaction between CRMP2 and α-tubulin, Co-immunoprecipitation was performed using an anti-CRMP2 antibody to precipitate α-tubulin and an anti-α-tubulin antibody to precipitate CRMP2. Both types of experiments confirmed the interaction between CRMP2 and α-tubulin in the hippocampus of rats. First, anti-CRMP2 was used for immunoprecipitation, and anti-α-tubulin was used for western blotting. Compared with that in the control group, the CRMP2-α-tubulin interaction was decreased in the hippocampus of the CUMS group, and it was increased after fluoxetine treatment (*F* = 4.92, *P* < 0.05, Figure 5A). Similarly, anti-α-tubulin was used for immunoprecipitation, and anti-CRMP2 was used for western blot. The α-tubulin-CRMP2 interaction was comparatively weaker in the CUMS group (*F* = 5.03, *P* < 0.05, Figure 5B).

### Effect of Microinjecting 5-aza Into the Hippocampal CA1 Region on Depressive-Like Behaviors

**Figure 6:**
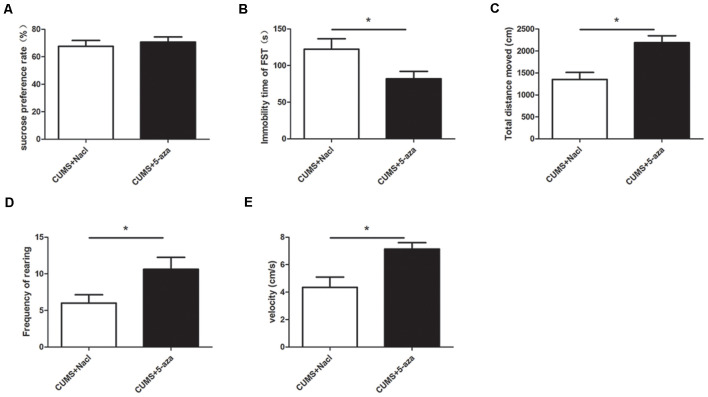
Effect of microinjecting 5-aza into the hippocampal CA1 region on depressive-like behaviors: sucrose preference rate in the SPT **(A)**, immobility time in the FST **(B)**, total distance traveled **(C)**, rearing frequency **(D)**, and velocity **(E)** in the OFT. *P < 0.05.

The SPT, OFT, and FST were used to evaluate the effects of microinjecting 5-aza into the CA1 region of the hippocampus on depressive-like behaviors. The results showed that compared with those of the rats in the CUMS + Nacl group, the distance traveled, rearing frequency, and velocity of the rats in the CUMS + 5-aza group were remarkably increased (distance traveled: *t* = −3.75, *P* < 0.05, Figure 6C; rearing frequency: *t* = −2.31, *P* < 0.05, Figure 6D; velocity: *t* = −3.19, *P* < 0.05, Figure 6E), and the immobility time in the FST of the rats of the CUMS + 5-aza group was significantly decreased (*t* = 2.30, *P* < 0.05, Figure 6B). However, no significant difference was observed in the sucrose preference of the rats in the CUMS + 5-aza group, although it tended to increase (*t* = −0.55, *P* > 0.05, Figure 6A).

### Effect of 5-aza Administration on Global DNA Methylation in the CA1 Region of the Hippocampus

**Figure 7:**
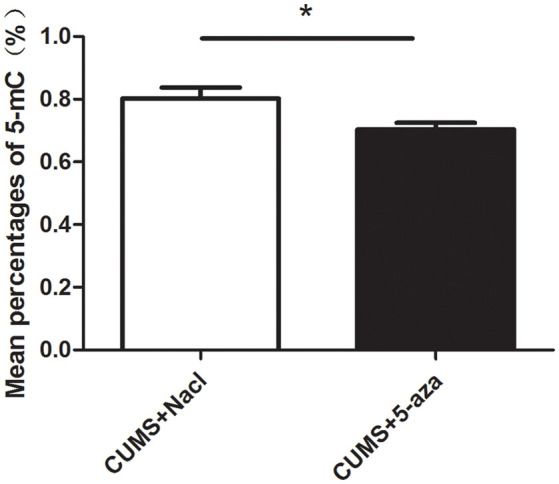
Effect of 5-aza administration on global DNA methylation in the CA1 region of the hippocampus. *P < 0.05.

The percentages of 5-mC of total DNA were measured as an indication of global DNA methylation levels. The 5-mC ELISA test was used to evaluate the effect of 5-aza treatment on global DNA methylation levels in the hippocampal CA1 region of rats. The results showed that microinjecting 5-aza into the CA1 region of the hippocampus significantly reduced the percent levels of global DNA methylation (*t* = 2.39, *P* < 0.05, Figure 7).

### Effect of 5-aza Treatment on CRMP2, pCRMP2, and α-Tubulin Isoform Expression

**Figure 8:**
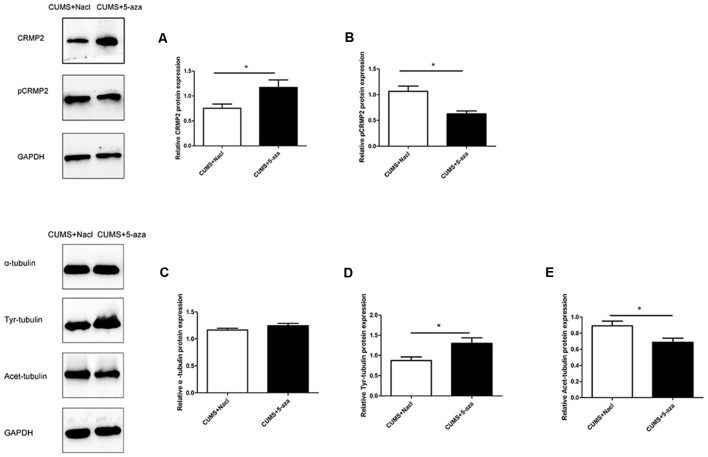
Effect of 5-aza treatment on CRMP2 **(A)**, pCRMP2 **(B)**, and α-tubulin (α-tubulin: **C**; Tyr-tubulin: **D**; Acet-tubulin: **E**) isoform expression. *P < 0.05.

Compared with those in the rats in the CUMS + Nacl group, CRMP2 protein expression was increased and pCRMP2 protein expression was decreased in the hippocampal CA1 region of the rats in the CUMS + 5-aza group (CRMP2: *t* = −2.44, *P* < 0.05, Figure 8A; pCRMP2: *t* = 3.62, *P* < 0.05, Figure 8B). Additionally, 5-aza treatment increased Tyr-Tubulin protein expression and decreased Acet-tubulin protein expression in the hippocampal CA1 region of the rats (α-tubulin *t* = 1.543, *P* > 0.05, Figure 8C; Tyr-Tubulin: *t* = −2.59, *P* < 0.05, Figure 8D; Acet-tubulin: *t* = 2.65, *P* < 0.05, Figure 8E).

### Effect of 5-aza Treatment on the Interaction Between CRMP2 and α-Tubulin

**Figure 9:**
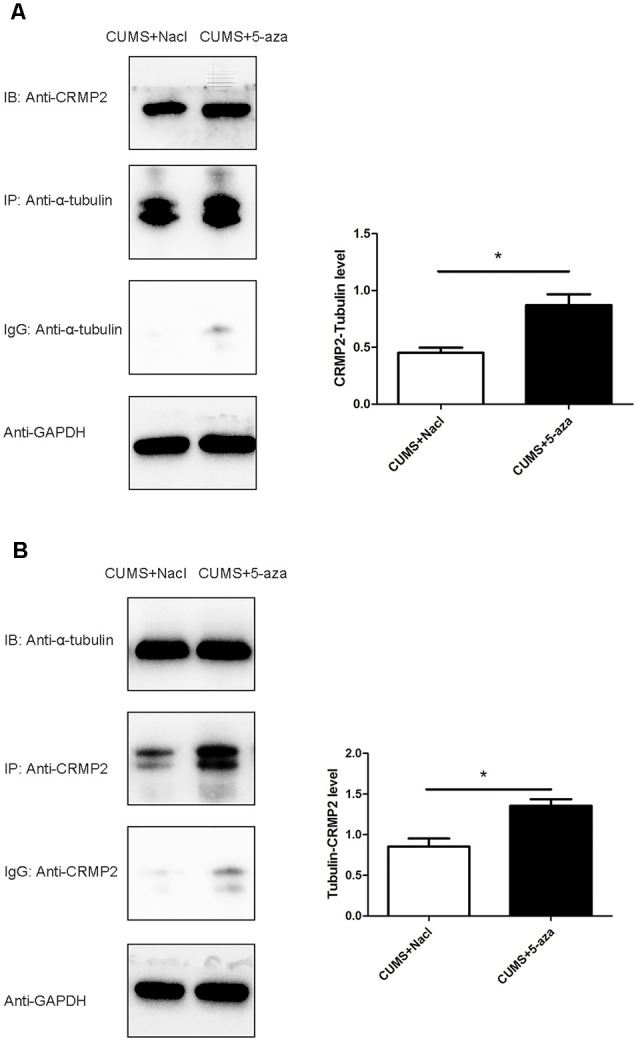
Effect of 5-aza treatment on the interaction between CRMP2 and α-tubulin **(A,B)**. *P < 0.05.

Co-immunoprecipitation was performed to assess the interaction between CRMP2 and α-tubulin in the hippocampal CA1 region of the 5-aza-treated rats. The CRMP2-α-tubulin interaction was also observed in the hippocampal CA1 region of the rats. The results showed that the α-tubulin band was detected in the immunoprecipitate of the anti-CRMP2 antibody. Compared with that in rats in the CUMS + Nacl group, the CRMP2-α-tubulin interaction was increased in the hippocampal CA1 region of the rats in the CUMS + 5-aza group (*t* = −4.00, *P* < 0.05, Figure 9A). Similarly, a CRMP2 band was detected in the immunoprecipitate of the anti-α-tubulin antibody. The α-tubulin-CRMP2 interaction also increased after 5-aza administration in the hippocampal CA1 region of rats (*t* = −3.97, *P* < 0.05, Figure 9B).

## Discussion

The CUMS rat model has been widely used to study mechanisms of depressive-like behaviors and actions of antidepressants. The SPT was used to assess anhedonia, which is one of the core symptoms of depression (Anisman and Matheson, 2005). The OFT was performed to assess general locomotor activity. Immobility time in the FST was used to evaluate the symptom of helplessness in a rat model of depression (Paré, 1992). In this study, the rat model of depression was successfully established by exposure to CUMS for 4 weeks. The rats exposed to CUMS showed reduced sucrose intake in the SPT, reduced distance traveled, rearing frequency and velocity in the OFT, and longer immobility time in the FST, which were similar to results from previous studies (Ayuob, 2017; Liu et al., 2018). Chronic fluoxetine treatment reversed the reduction in sucrose preference in the SPT, distance traveled, rearing frequency and velocity in the OFT, and increased immobility time in the FST, which indicated that fluoxetine has antidepressant-like effects.

In the present study, we investigated the effect of CUMS and fluoxetine treatment on the expression of DNMTs in the hippocampus of rats. DNA methylation in mammals is catalyzed by three main DNMTs: DNMT1, DNMT3a, and DNMT3b. We observed an upregulation of DNMT1 and DNMT3a in the hippocampus of stressed rats, and fluoxetine treatment significantly downregulated the expression of DNMT1 and DNMT3a. We further explored the CRMP2 expression and DNA methylation levels in the hippocampus of rats. Our data showed a downregulation of the CRMP2 protein levels in the hippocampus of stressed rats, and fluoxetine treatment upregulated the CRMP2 protein levels. This finding proves the results of previous publications, which showed a decrease in CRMP2 in the hippocampus of an animal model of depression (Carboni et al., 2006). Furthermore, we initially analyzed the CpG island DNA methylation levels in the CRMP2 promoter region, and the results showed that the rate of DNA methylation in the CpG island of the CRMP2 promoter region in the hippocampus of stressed rats was significantly higher than that in the hippocampus of control rats. Fluoxetine treatment significantly decreased the CpG island DNA methylation levels in the CRMP2 promoter region. Previous studies have suggested that the silencing or low expression of genes is due to CpG island DNA methylation, which is associated with high DNMTs expression (Tammen et al., 2013). Therefore, our results demonstrated that DNMT1 and DNMT3a regulate the level of DNA methylation in the CRMP2 gene promoter region, thus affecting the expression of CRMP2 in the hippocampus of rats and participating in the pathogenesis of depression.

Current evidence supports the idea that CRMP2 contributes to the etiology of depression. However, the regulatory mechanisms underlying the role of CRMP2 remain unclear. CRMP2 is involved in neuronal migration, axonal growth, dendritic development, and synaptic plasticity (Ip et al., 2014). The cytoskeletal microtubular system is essential for the remodeling and extension of axons and dendrites (Bianchi et al., 2005a). CRMP2 acts as a signaling phosphoprotein that modulates cytoskeletal organization and participates in neurite formation and axonal outgrowth. The phosphorylation of CRMP2 might change its binding partners and functions (Zhu et al., 2010). The dynamics of the cytoskeletal microtubular system are strictly correlated with neuronal plasticity (Bianchi et al., 2005b). Therefore, in the present study, we analyzed the functions of CRMP2 in the cytoskeletal microtubular system. The results showed that exposure to CUMS for 4 weeks induced a decrease in CRMP2 expression and an increase in pCRMP2 expression in the hippocampus of rats, and chronic fluoxetine treatment reversed these effects on the expression of CRMP2 and pCRMP2. Additionally, the co-immunoprecipitation results showed that CRMP2 formed a complex with α-tubulin. Compared with that in the control group and fluoxetine group, the CRMP2-α-tubulin interaction in the CUMS group was comparatively weaker. We further examined microtubule dynamics by analyzing Acet-tubulin and Tyr-tubulin. Together with previous findings (Bianchi et al., 2003), our data showed that exposure to CUMS for 4 weeks decreased the expression of Tyr-tubulin, which is associated with the dynamic form of microtubules; these results indicated decreased microtubule dynamics. Chronic fluoxetine treatment decreased the stable form of microtubule Acet-tubulin, suggesting that fluoxetine could be effective in rescuing microtubule stabilization in the hippocampus of stressed rats. These data suggest that CRMP2 is involved in the pathogenesis of depression by regulating the cytoskeletal microtubule system.

Environmental stress was shown to upregulate the expression of specific DNMTs in the hippocampus and to change the expression of depression-related candidate proteins (Menke and Binder, 2014). In the present study, stressed rats that exhibit depressive behaviors showed an upregulation of the DNMT1 and DNMT3a proteins in the hippocampus. Considering the susceptibility of the hippocampal CA1 region to stress, we investigated the involvement of DNA methylation in the regulation of CUMS-induced depressive-like behaviors and the underlying mechanisms by injecting 5-aza into the hippocampal CA1 regions of rats. In our study, microinjecting 5-aza into the hippocampal CA1 region effectively reduced global DNA methylation, suggesting that 5-aza treatment effectively altered the DNA methylation status and might be used in subsequent studies. Additionally, injection of 5-aza into the hippocampal CA1 region of rats induced antidepressant-like effects, including increased distance traveled, rearing frequency and velocity in the OFT, and decreased immobility time in the FST. Our findings supported the possibility that DNA methylation in the hippocampal CA1 region is crucial for the development of depressive-like behaviors.

Previous data indicate that an increase in DNA methylation at specific genomic sites may reduce gene expression (Boyes and Bird, 1992). These genes may play an important role under stress conditions and, therefore, predispose to the development of stress-induced depressive-like behaviors. In the present study, 5-aza treatment not only exerted antidepressant-like effects and reduced DNA methylation but also significantly increased CRMP2 protein expression in the hippocampal CA1 region, further supporting the notion that DNA methylation-dependent CRMP2 gene expression is involved in the pathogenesis of depression. Additionally, 5-aza treatment increased Tyr-Tubulin protein expression and decreased Acet-tubulin protein expression in the hippocampal CA1 regions of rats, indicating that 5-aza could increase microtubule dynamics. The α-tubulin-CRMP2 interaction also increased after 5-aza injection into the hippocampal CA1 regions of rats. These results further suggest that the DNA methylation-mediated regulation of CRMP2 is indicative of its role in the cytoskeletal microtubule system in depression.

In recent years, there has been increasing interest in the correlation between depression and cerebrovascular disease. Cerebral infarction increases the incidence of post-stroke depression, with increased morbidity and mortality, which has become a common and acute disease. Several studies have reported the changes in CRMP2 expression during the process of ischemia (Chen et al., 2007; Yang et al., 2016). Proteomic technology has revealed elevation of CRMP2 protein expression in the ischemic brain of rats (Koh, 2011). In a rodent study, the result found higher BDNF promoter IV methylation levels in the hippocampus of post-stroke depression (Jin et al., 2017). Based on these findings, it would be worth exploring the underlying mechanisms of CRMP2 DNA methylation in the pathological processes of post-stroke depression.

Taken together, our results identified a crucial role of CRMP2 in the pathogenesis of depression. These observations implicate DNA methylation in the molecular mechanisms that control stress-induced depressive-like behaviors and could ultimately lead to the development of improved treatment for depression. However, some questions remain in this study. As a broad-spectrum inhibitor of DNA methylation, 5-aza lacks gene-specific effects. Further studies using inhibitors of gene-specific DNA methylation are necessary to explore the role of CRMP2 in depression. Additionally, we did not verify our findings *in vitro* in this study, and an *in vitro* cell model should be established in future studies.

## Data Availability Statement

The original contributions presented in the study are included in the article, further inquiries can be directed to the corresponding authors.

## Ethics Statement

The animal study was reviewed and approved and all the procedures and animal experiments of this study were performed in agreement with the guidelines outlined in the legislation of the P.R. China regarding the ethical use of laboratory animals and approved by the Institutional Animals Care Committee of Renmin Hospital of Wuhan University.

## Author Contributions

ZL was responsible for funding acquisition. GW and ZL designed and supervised the study. DX and SS carried out the experimental procedures and analyzed the date. ZL and DX interpreted results of experiments and drafted the manuscript. GW and ZL revised the manuscript. All authors provided feedback on manuscript. All authors contributed to the article and approved the submitted version.

## Conflict of Interest

The authors declare that the research was conducted in the absence of any commercial or financial relationships that could be construed as a potential conflict of interest.
